# Meta-analysis of clinical efficacy of electroacupuncture versus conventional treatment for postoperative urinary retention in cervical cancer

**DOI:** 10.1097/MD.0000000000035580

**Published:** 2023-10-27

**Authors:** Jinlang Tan, Rui Gong, Qian Zhang, Yi Zheng, Le Ma, Shuai Shi

**Affiliations:** a Heilongjiang University of Traditional Chinese Medicine, Harbin, Heilongjiang, China; b The Second Hospital Affiliated to Heilongjiang University of Traditional Chinese Medicine, Harbin, Heilongjiang, China.

**Keywords:** acupuncture, cervical cancer, electroacupuncture, meta-analysis, needling, postoperative cervical cancer, urinary retention

## Abstract

**Background:**

To conduct a meta-analysis on the clinical efficacy of electroacupuncture in the treatment of postoperative urinary retention in cervical cancer, and to provide a theoretical basis for the promotion of electroacupuncture in the treatment of this disease.

**Methods:**

Computer searches of the Cochrane library, Web of science, PubMed, Embase, Chinese Biomedical Literature Database, Wanfang database, Wipu database, and China National Knowledge Infrastructure Database database were conducted to find randomized controlled trials on electroacupuncture for postoperative urinary retention recovery in cervical cancer, all from the time of database creation to October 2022. Two evaluators independently evaluated the quality of the included literature and extracted the data. Data were combined and analyzed using RevMan 5.4.

**Results:**

A total of 21 Randomized controlled trials with 1532 patients, 789 in the treatment group and 743 in the control group, were included. One descriptive analysis was performed and 20 Meta-analyses were performed. Meta-analysis results showed that: The electroacupuncture group was more effective than the control group in promoting recovery from urinary retention after cervical cancer, with a statistically significant difference [relative risk (RR)] = 1.32, 95% confidence interval (CI 1.26, 1.39), *P* < .00001; The duration of indwelling catheterization was reduced in the electroacupuncture group compared with the control group, with a statistically significant standard mean difference = −1.43, 95% CI (−1.62, −1.24), *P* < .00001; The healing rate in the electroacupuncture group was higher than that in the control group, with a statistically significant difference [RR] = 1.92, 95% CI (1.59, 2.30), *P* < .00001; The rate of urinary tract infection in the electroacupuncture group was lower than that in the control group, with a statistically significant difference [RR] = 0.22, 95% CI (0.10, 0.45), *P* < .00001. The incidence of urinary retention was lower in the electroacupuncture group than in the control group, and the difference was statistically significant [RR = 0.26, 95% CI (0.18, 0.39), *P* < .01].

**Conclusion:**

Electroacupuncture can promote the recovery of urinary retention after cervical cancer surgery, and can improve the healing rate of patients after surgery, reduce the occurrence of urinary tract infection and shorten the duration of indwelling catheterization.

## 1. Introduction

Cervical cancer, also known as uterine cervix cancer, is a malignant tumor that occurs in the epithelium of the vaginal part of the uterine cervix and the cervical canal. The pathologic type of cervical cancer is mainly squamous carcinoma, which accounts for about 80%, and adenocarcinoma accounts for about 15% to 20%.^[1]^ In 2020, there were about 604,000 new cases of cervical cancer worldwide, including nearly 110,000 new cases and 47,700 deaths in China.^[2]^ The treatment of choice for early-stage cervical cancer is radical cervical cancer surgery,^[3]^ but the most common complication is postoperative UR (UR).^[4]^Postoperative UR is a condition in which the bladder fills with urine after surgery but does not pass it on its own or passes urine on its own but with a residual urine of ≥ 100 mL, which can lead to overstretching of the bladder and permanent detriment to the forced urethral muscle, and is considered to be a common complication of surgery and anesthesia.^[4]^ The routine clinical management is indwelling catheterization for 7 to 14 days,^[5]^ but long-term indwelling catheterization increases the patient’s pain, raises the rate of urinary tract infection,^[6]^ affects postoperative recovery, and is a heavy physical, psychological and financial burden on the patient.^[7]^Acupuncture is an ancient form of Chinese medicine in which fine needles are inserted into the skin at precise points (acupoints) to balance the flow of energy (qi) throughout the body. Electroacupuncture, the application of electrical stimulation to acupuncture needles, is a combination of acupuncture and electrophysiological effects that increases sensation of acupuncture and reduces the amount of work involved in rotating the needles.^[8]^ Electroacupuncture is an effective method for reducing the incidence of UR and urinary tract infections and shortening the length of hospital stay.^[9]^ Numerous studies have shown that acupuncture modulates neurotransmitters and objectively improves urodynamics.^[10,11]^ However, acupuncture has not yet been included in the treatment guidelines by the European Association of Urology and the American Urological Association.^[5,12]^ Therefore, a meta-analysis was performed to provide recommendations for clinical decision making.

## 2. Information and methods

### 2.1. Search method

Computerized searches were conducted across various databases, including Cochrane library, Web of science, PubMed, Embase, Chinese Biomedical Literature Database, Wanfang database, Wipro database, and China National Knowledge Infrastructure Database database, to identify relevant research literature on the use of electroacupuncture in promoting the recovery of UR after radical surgery for cervical cancer. The search timeframe for all databases ranged from their establishment to October 2022. Additionally, the references of included literature were traced.

Both subject and free words were used as search terms in English, such as acupuncture, cervical cancer, UR, uterine cervical neoplasms, acupuncture therapy, electroacupuncture, and electric. The Chinese search terms included “cervical cancer, postoperative cervical cancer, UR, acupuncture, electroacupuncture, needling”. To ensure a systematic and comprehensive search, a combined search of subject words and free words was performed across different databases. The search formula is shown in Table 1

**Table 1:** PubMed search strategy.

No.	Search strategy
#1TS	(Acupuncture) OR (Acupuncture therapy) OR (Acupuncture treatment) OR (Acupuncture treatments) OR (Treatment, Acupuncture) OR (Therapy, Acupuncture) OR (Pharmacoacupuncture treatment) OR (Treatment, Pharmacoacupuncture) OR (Pharmacoacupuncture Therapy) OR (Therapy, Pharmacoacupuncture) OR (Acupotomy) OR (Acupotomies) OR (Electroacupuncture)
#2TS	(Cervix cancer) OR (Cervical Neoplasm, Uterine) OR (Cervical Neoplasms, Uterine) OR (Neoplasm, Uterine Cervical) OR (Neoplasms, Uterine Cervical) OR (Uterine Cervical Neoplasm) OR (Neoplasms, Cervical) OR (Cervical Neoplasms) OR (Cervical Neoplasm) OR (Neoplasm, Cervical) OR (Neoplasms, Cervix) OR (Cervix Neoplasms) OR (Cervix Neoplasm) OR (Neoplasm, Cervix) OR (Cancer of the Uterine Cervix) OR (Cancer of the Cervix) OR (Cervical Cancer) OR (Uterine Cervical Cancer) OR (Cancer, Uterine Cervical) OR (Cancers, Uterine Cervical) OR (Cervical Cancer, Uterine) OR (Cervical Cancers, Uterine) OR (Uterine Cervical Cancers) OR (Cancer of Cervix) OR (Cervix Cancer) OR (Cancer, Cervix) OR (Cancers, Cervix) AND (urinary retention) OR (Retention, Urinary)
#3TS	(Urinary retention)
#4TS	#3 AND #2 AND #1

OR = odds ratio.

### 2.2. Inclusion and exclusion criteria

#### 2.2.1. Inclusion criteria.

The study included the following criteria:

1. Study type: Randomised controlled trials, with language limited to English and Chinese.
2. Study subjects: Patients with UR after cervical cancer surgery who were clearly diagnosed, regardless of race or nationality.
3. Intervention: The control group was given conventional treatment, conventional rehabilitation, conventional nursing care, intermittent catheterization technique, and other conventional methods. The experimental group was treated with electroacupuncture on the basis of the control group, with unlimited acupoints and duration of treatment.
4. Outcome indicators: The main indicators were the treatment efficiency, incidence of UR, and duration of indwelling catheterization of electroacupuncture. The secondary indicators were the recovery rate and urinary tract infection rate to assess the efficacy of electroacupuncture.

#### 2.2.2. Exclusion criteria.

The following types of literature were excluded from the study:

1. Literature where the main treatment method was a non-acupuncture therapy.
2. Literature where different acupuncture techniques were compared or where herbal treatments were utilized, or where only moxibustion was used.
3. Non-randomized trials, quasi-experimental and observational studies, animal studies, qualitative studies, letters, news articles, editorials and reviews were also excluded.
4. Systematic evaluations or meta-analyses related to the subject of this study.

### 2.3. Literature screening and data extraction

The literature was screened independently by 2 researchers according to the inclusion and exclusion criteria, and cross-checked. Articles were first read in the title and abstract to exclude those that did not meet the criteria. Then, the articles that met the inclusion criteria were further read in full, and those that did not were excluded. In case of disagreement, the decision to include was made through discussion.

The following data were extracted from each selected study: first author, year of publication, sample size, details of the intervention and control groups, and outcome indicators. If study data were insufficient or ambiguous, the evaluator contacted the corresponding author by email for more information.

### 2.4. Quality assessment

The quality of the included literature was assessed according to the Cochrane 2011 update of the systematic reviewer’s handbook.^[13]^ The assessment criteria included the generation of the randomization order, concealment of the random allocation scheme, blinding of study subjects and intervention implementers, blinding of outcome measures, completeness of data for outcome indicators, potential for selective reporting of study results, and other aspects of bias.

Each project was evaluated independently by 2 researchers, and in the event of inconsistent results, the decision was discussed and adopted by the 2 researchers together. If this was not possible, consultation with a third researcher with a senior title was sought to resolve any discrepancies.

### 2.5. Data analysis

Meta-analysis was performed using RevMan 5.4 software (https://revman.cochrane.org/). Included studies were analyzed in subgroups according to differences in interventions to minimize clinical heterogeneity, in addition to overall meta-analysis. Relative risk (RR) values and their 95% confidence intervals (CIs) were used as effect analysis statistics for dichotomous variables, with a test level of a = 0.05. Information on continuous variables was expressed as mean difference or standard mean difference, and 95% CIs were calculated for each effect size. Statistical heterogeneity between studies was tested using the *I*^2^ test or *P* value.

If *P* > .1 and *I*^2^ < 50%, this indicated fair heterogeneity, and a fixed effects model was selected for meta-analysis. If *P* ≤ .1 and *I*^2^ ≥ 50%, this indicated large heterogeneity, and the source of heterogeneity was explored before meta-analysis using a random-effects model with subgroup analysis or sensitivity analysis. Publication bias was assessed using funnel plot analysis if a sufficient number of trials were included (10 trials). If fewer than ten studies were included, publication bias was not assessed.

## 3. Results

### 3.1. Literature search results

**Figure 1.**
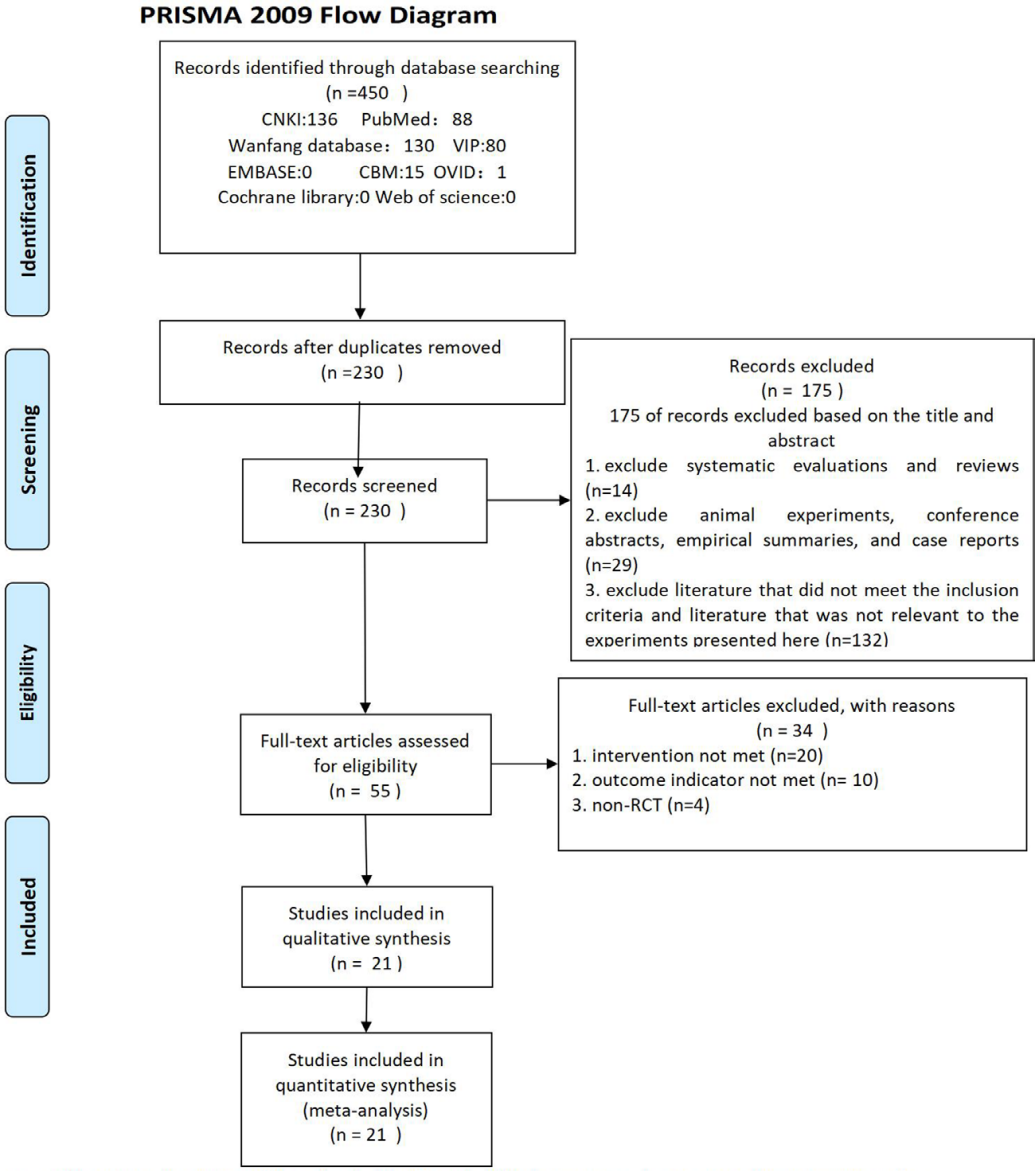
Flow chart of literature search.

A total of 450 articles were searched according to our search strategy. After removing duplicates and filtering titles and abstracts, we obtained 55 full-text articles. Based on exclusion criteria, 21^[14–34]^ articles were entered into the final analysis. There was no statistical difference between the general information of patients in all literature control and treatment groups (*P* > .05). The literature screening process and results are shown in Figure 1.

### 3.2. Characteristics of the included literature

The basic characteristics of the included literature are shown in Table 2. Although we did not set country restrictions, all 21 studies were conducted in China and published between 2003 and 2021. We included 21 studies, 10 of which^[15,17,20,21,23–26,28,33]^ had a duration of less than or equal to 10 days and 11 of which^[14,16,18,19,22,27,29–32,34]^ had a duration of more than 10 days. All included studies used electroacupuncture in combination with or without other treatments as an intervention, with 15 studies^[14–21,24,27–31,34]^ compared electroacupuncture with usual care and physiotherapy and 6 studies^[22,23,25,26,32,33]^ compared electroacupuncture with pharmacological treatment. Seven studies^[14,18,20,24,27,29,30]^ set up electroacupuncture as an intervention, while the control group had no intervention. Each study reported comparable baseline characteristics between groups.

**Table 2:** Basic information of included literature

Inclusion in the study	Inclusion (T/C)	Article type	Intervention time	Treatment group	Control group	Outocmes
Ye TY 2021^[14]^	32/32/33⑥	RCT	84 d	EA + RC	RC	①③
Yang DR 2019^[15]^	36/36	RCT	10 d	EA + FT + RC	FT + RC	①③④⑤
Hu XF 2015^[16]^	42/42	RCT	14 d	EA + RC	RC	①⑤
Yang M 2015^[17]^	30/30	RCT	10 d	EA + FT + RC	FT + RC	①③④⑤
Ye PZ 2014^[18]^	74/33	RCT	14 d	EA + RC	RC	①③④
Mou SL 2012^[19]^	26/32	RCT	14 d	EA + FT + RC	RC	①④⑤
Zhou X 2010^[20]^	35/33	RCT	5 d	EA + RC	RC	①②
Qiu XL 2010^[21]^	31/31	RCT	5 d	EA + FT + RC	FT + RC	①⑤
Li XM 2009^[22]^	60/60	RCT	15 d	EA + FT + RC	N + RC	①
Peng HD 2009^[23]^	23/23	RCT	5 d	EA + RC + FT	N + RC + FT	①②
Chen XY 2008^[24]^	45/39	RCT	10 d	EA + RC	RC	①⑤
Chen XN 2006^[25]^	30/30	RCT	5 d	EA + RC	N + RC	①②
Zhou YM 2003^[26]^	40/34	RCT	5 d	EA + RC	N + RC	①②
Chen XN 2020^[27]^	37/37	RCT	12 d	EA + RC	RC	①③④⑤
Sun SF 2018^[28]^	42/42	RCT	10 d	EA + CT	CT	①②
Dong Y 2018^[29]^	40/40	RCT	12 d	EA + RC	RC	①③④⑤
Liu YM 2017^[30]^	19/22	RCT	21 d	EA + RC	RC	①③④⑤
Peng Y 2019^[31]^	32/32	RCT	30 d	EA + RC + FT	FT + RC	①②③④⑤
Yi WM 2011^[32]^	40/40	RCT	21 d	EA + RC	Vitamin B12 + RC	①⑤
Li Y 2003^[33]^	52/52	RCT	7 d	EA	N	①②
Yi WM 2008^[34]^	55/55	RCT	28 d	EA + RC + TDP	RC + TDP	①

T is the intervention group; C is the control group. ①Effective rate; ②Cure rate; ③Urinary tract infection; ④Time of indwelling catheter; ⑤Incidence of urinary retention; ⑥32/32/33 represent the number of cases in the control group, acupuncture group 1, and acupuncture group 2, respectively.

EA = Electroacupuncture, RCTs = randomized controlled trials.

### 3.3. Methodological quality of the included studies

The principle of random allocation was adopted in all 21^[14–34]^ included publications. However, 11^[15,16,19–24,30,31,33]^ clinical trials did not specify the method of random allocation and were therefore rated as unknown risk. Seven^[14,17,27–29,32,34]^ articles used the random number table method for randomization, 1^[18]^ used SAS 6.0 software (https://www.sas.com/zh_cn/home.html) for randomization, and 2^[25,26]^ articles used the coin toss method for randomization and were rated as low risk. Allocation concealment was not specified in any of the literature and was therefore rated as unknown risk.

None of the included literature controls used a placebo intervention, so all literature was high risk for blinded use. However, some literature used blinding for study evaluation and data statistics. Two^[22,34]^articles reported only treatment efficacy rates, resulting in selective reporting and a high-risk rating. All included literature had complete information and no conflicts of interest, resulting in a low-risk rating.

### 3.4. Risk of literature bias assessment

**Figure 2.**
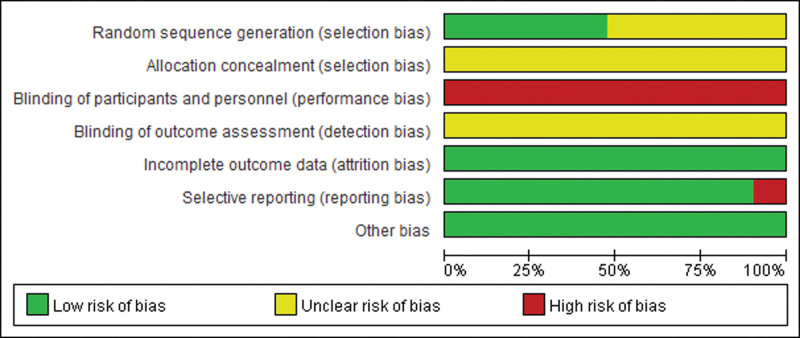
Evaluation of the risk of literature bias.

**Figure 3.**
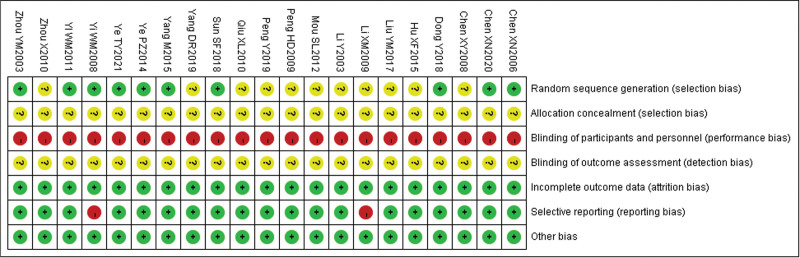
Evaluation of the risk of literature bias.

A literature risk of bias assessment was conducted on the literature. The results of the literature bias risk assessment are shown in Figure 2 and Figure 3.

### 3.5. Meta-analysis results

#### 3.5.1. Efficacy.

**Figure 4.**
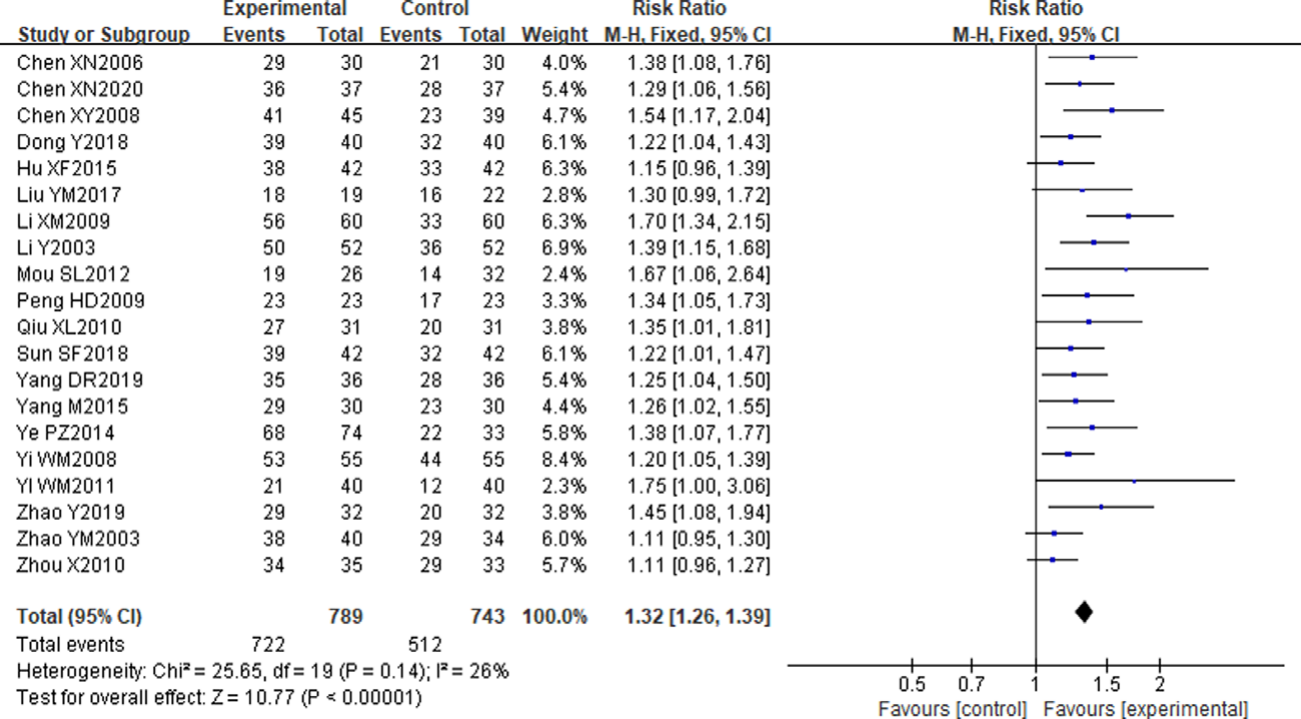
Comparison of total clinical efficiency of electroacupuncture for postoperative urinary retention in cervical cancer.

A descriptive analysis was performed as there was significant clinical heterogeneity in the duration of intervention (84 days) in one of the studies, Ye Sun^[14]^ 2021, compared to the other studies. Meta-analysis was performed on 20^[15–34]^ studies with intervention times ranging from 5 to 30 days. The combined sample size was 1532 cases, 789 cases in the treatment group and 743 cases in the control group. Twenty studies had slightly different nomenclature for each class, and by carefully reading the original evaluation criteria in the included literature, the data were extracted and named as “cured The effective rate of treatment was calculated by specifying that the effective rate = (number of cured + number of apparently effective + number of effective)”/(total number. number)/total number. Meta-analysis showed that the treatment effectiveness rate was higher in the electroacupuncture group than in the control group, with a statistically significant difference [RR] = 1.32, 95% CI (1.26, 1.39), *P* < .00001. see Figure 4.

#### 3.5.2. Incidence of UR.

**Figure 5.**
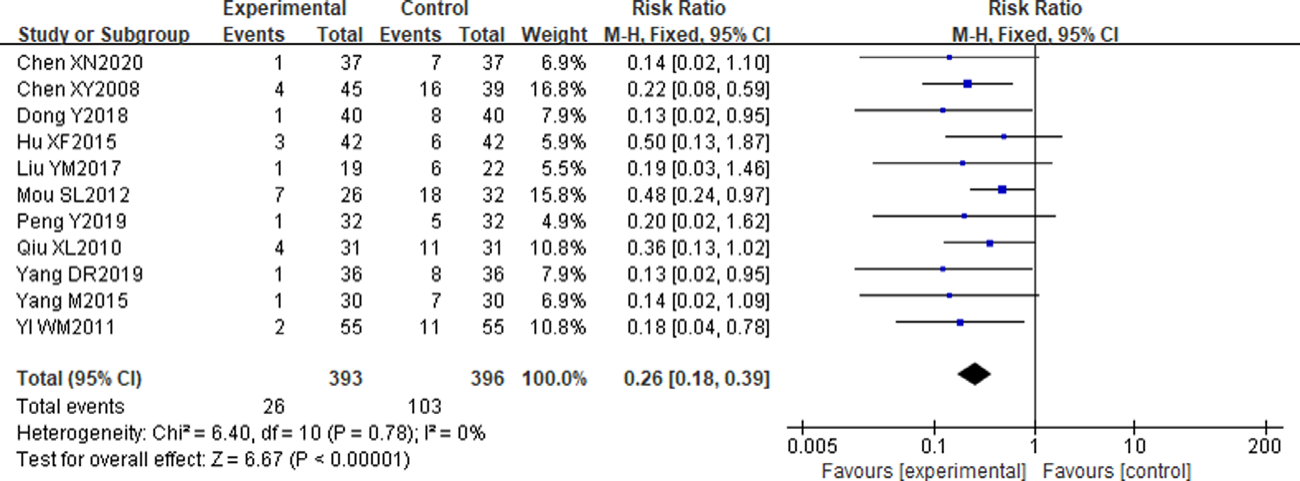
Comparison of the incidence of urinary retention after electroacupuncture for cervical cancer.

Eleven^[15–17,19,21,24,27,29–32]^ studies reported a comparison of the incidence of UR after treatment. The results of the heterogeneity test suggested no heterogeneity in the included literature (*P* = .78, *I*^2^ = 0%) and were analyzed using a fixed effects model. The results showed that the incidence of UR was lower in the electroacupuncture group than in the control group, with a statistically significant difference [RR = 0.26, 95% CI (0.18, 0.39), *P* < .01]. See Figure 5.

#### 3.5.3. Comparison of duration of catheterization placement.

**Figure 6.**
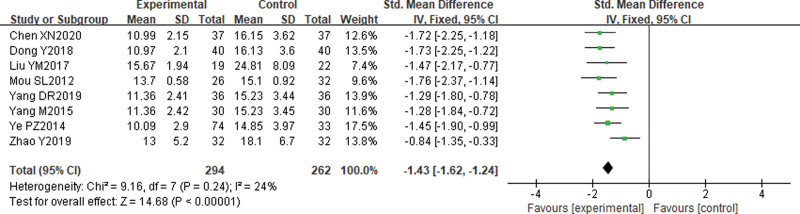
Comparison of the duration of indwelling catheterization after electroacupuncture for cervical cancer.

Eight^[15,17–19,27,29–31]^ studies reported comparisons of the duration of indwelling catheterization after treatment. Meta-analysis results showed that the duration of indwelling catheterization was shorter in the electroacupuncture group than in the control group [Standard mean difference = −1.43, 95% CI (−1.62, −1.24), *P* < .00001]. See Figure 6.

#### 3.5.4. Healing rates.

**Figure 7.**
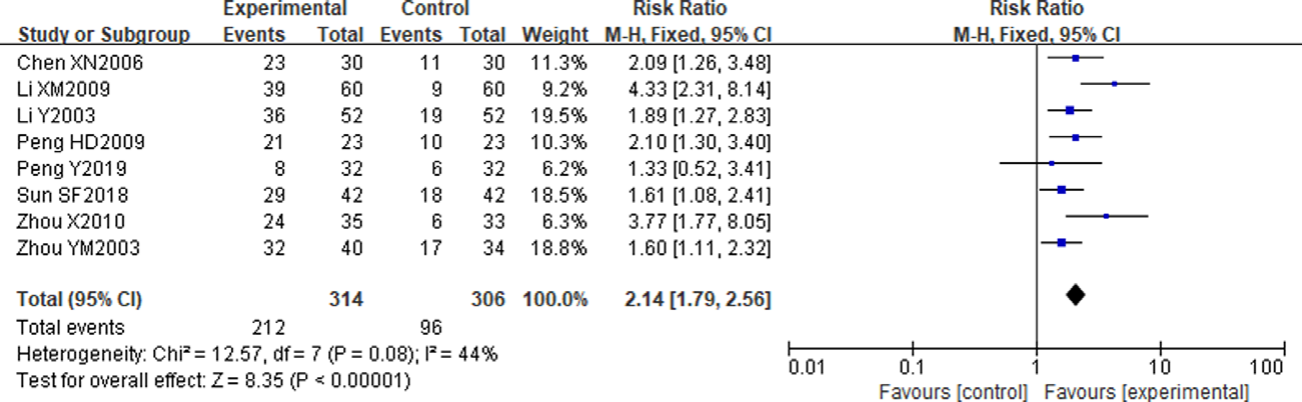
Comparison of healing rates after electroacupuncture for cervical cancer.

Eight^[20,22,23,25,26,28,31,33]^ studies reported comparative healing rates after treatment. The results of the heterogeneity test suggested low heterogeneity between the included studies (X2 = 5.52, *P* = .08, *I*^2^ = 44%), so a fixed effects model was chosen. the results of the Meta-analysis showed that the healing rate was higher in the electroacupuncture group than in the control group [RR = 2.14, 95% CI (1.79, 2.56)], *P* < .00001. see Figure 7.

#### 3.5.5. Rate of urinary tract infections.

**Figure 8.**
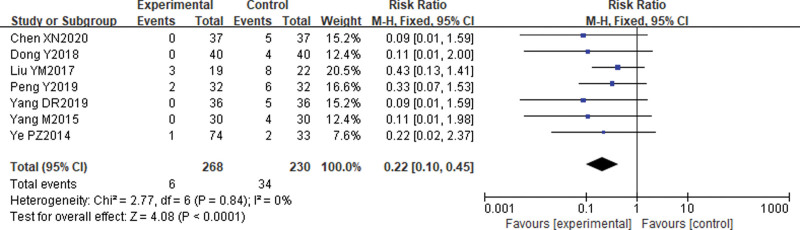
Comparison of urinary tract infection rates after electroacupuncture for cervical cancer.

Seven^[15,17,18,27,29–31]^ studies reported comparative rates of posttreatment urinary tract infections. The results of the heterogeneity test suggested that the difference in heterogeneity between the included studies was not statistically significant (X2 = 2.77, *P* = .84, *I*^2^ = 0%), so a fixed effects model was chosen. the results of the Meta-analysis showed that the rate of urinary tract infection was lower in the electroacupuncture group than in the control group [RR] = 0.22, 95% CI (0.10, 0.45), *P* < .00001. see Figure 8.

### 3.6. Results of descriptive analysis

A descriptive analysis was performed on the 1 paper for which Meta-analysis could not be performed. In the study by Ye Sun et al^[14]^ clinical efficacy was used and the rate of UR was rated on 3 scales including cured, effective and ineffective. The trial was divided into an electroacupuncture group and a control group. The results compared the efficiency of the 2 groups and showed that the electroacupuncture group was more effective than the control group (*P* < .05), which was consistent with Meta-analysis. In addition, a number of urodynamic parameters were assessed. In the pre- and posttreatment comparisons of residual urine volume, maximum urinary flow rate, voided urine volume, forced urinary muscle pressure at maximum urinary flow rate, maximum forced urinary muscle systolic pressure and maximum bladder capacity in a single group, the posttreatment results were found to be better than the pretreatment results, with statistically significant differences (all *P* < .05). All urodynamic parameters, except maximum bladder capacity, were statistically significantly different after treatment compared to the control group (all *P* < .05). This is generally consistent with the findings of Hu Hui et al,^[35]^ indicating the positive significance of electroacupuncture in improving bladder function.

### 3.7. Publication bias analysis

**Figure 9.**
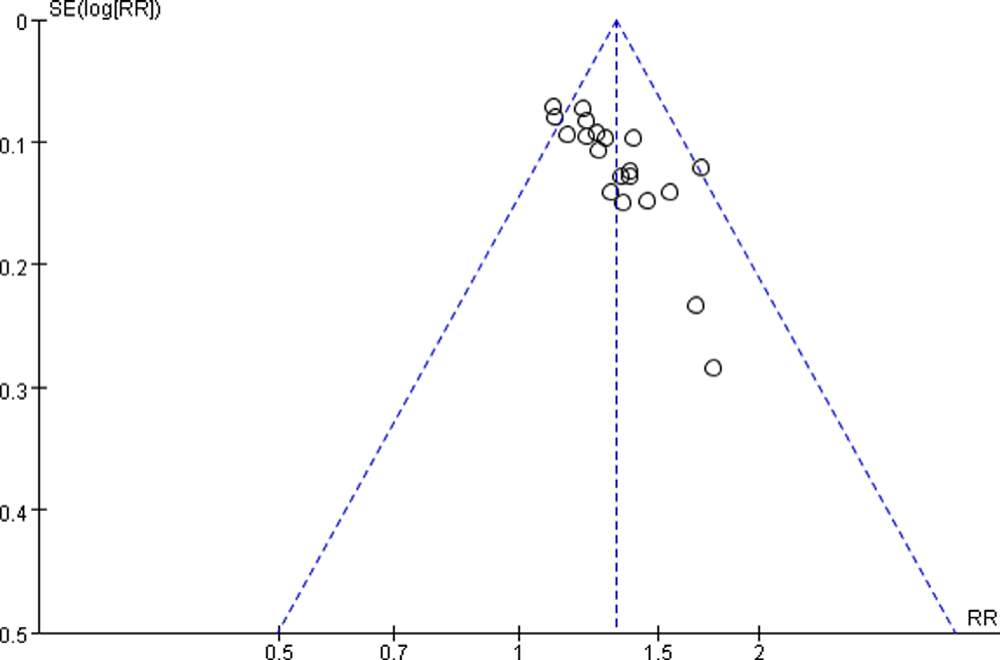
Meta-analysis funnel plot of the efficiency of electroacupuncture for postoperative urinary retention in cervical cancer.

The inverted funnel plot of publication bias was plotted using total clinical effectiveness as an indicator (see Fig. 9)., and the lack of symmetry in the plot suggests the possibility of publication bias.

## 4. Discussion

UR after radical surgery for cervical cancer is a common problem, and it can be difficult to achieve satisfactory outcomes with a single treatment modality. Bladder dysfunction is one of the primary causes of postoperative UR in patients. UR can cause unbearable distention and pain in the lower abdomen, as well as an inability to empty urine from the bladder.^[36,37]^ Dysfunction of the muscles or nerves that control voiding can interfere with normal bladder and ureteral function, leading to bladder dysfunction or failure of pelvic floor relaxation, resulting in UR.^[38,39]^

Electroacupuncture can bi-directionally regulate the pubic nerve-bladder reflex and urethral sphincter contraction, resulting in lower residual urine volume, higher initial urinary bladder capacity, maximum urinary flow rate, and maximum urinary bladder capacity. Electroacupuncture can also improve bladder compliance, promote recovery of urinary function, reduce bladder-related complications,^[40,41]^ and reduce symptoms such as pain by inhibiting the sympathetic nervous system.^[42]^ Acupuncture can adjust the function of the bladder from both central and peripheral perspectives, improving urethral dynamics.^[43,44]^ Electroacupuncture is often used clinically to relieve pain and obstructive symptoms in patients.

The results of the study show that electroacupuncture for postoperative UR in cervical cancer can effectively improve clinical outcomes and is simple to perform. The study found that the frequency of selecting 7 acupuncture points, namely Sanyinjiao, Fusanli, Zhongji, Guangyuan, Bladder Yu, Yinlingquan, and Doudao, was > 50%. This suggests that these 7 acupuncture points can be used as commonly prescribed acupuncture points for the treatment of postoperative UR after cervical cancer. The corresponding acupuncture points can then be selected in conjunction with the actual site of patient morbidity, which has high guiding significance for clinical practice.

Combining electroacupuncture with conventional treatment for UR during radical cervical cancer surgery has good clinical effects. While the catheter is routinely retained, clinical adjuncts are based on physiotherapy, comprehensive nursing interventions, and medication; TCM is based on acupuncture, massage, oral Chinese medicine, and acupressure.^[45]^ Therefore, electroacupuncture can be a valuable addition to conventional treatment methods for postoperative UR in cervical cancer patients.

## 5. Strengths and limitations of the study

While some systematic evaluations and meta-analyses have been conducted on the treatment of UR with acupuncture, there are currently no specific reports on the use of electroacupuncture to treat UR in patients with postoperative cervical cancer. The pathogenesis of UR due to postoperative cervical cancer is significantly different from other causes, making further studies on this topic necessary. Two systematic evaluations and meta-analyses of acupuncture for UR after radical cervical cancer surgery were conducted in 2014 and 2016, respectively.^[46,47]^ However, these studies did not focus specifically on electroacupuncture as a treatment modality and included a small amount of literature, had an insufficient sample size, and chose to include different interventions and outcome indicators than our study. One study^[48]^ reported on the role of acupuncture in promoting recovery after gynaecological surgery, but did not specifically focus on postoperative UR. Two studies^[49,50]^ reported a systematic evaluation and meta-analysis of acupuncture interventions for postpartum UR. However, the pathogenesis, recovery time, and difficulty of recovery for postpartum UR differ significantly from our research topic.

This study has some limitations, including a small number of included articles and a lack of high-quality, multicentre, clinically randomized controlled studies. Additionally, the study did not report in detail on randomization methods versus allocation concealment methods, which can cause selective bias. There was also a lack of adverse outcomes reported in the included literature. Furthermore, the number of included Chinese articles varied greatly in quality, indicating a need to expand the sample size and conduct long-term follow-up studies to obtain more conclusive evidence.

## 6. Safety analysis

During acupuncture treatment, patients experiencing dizziness, broken needles, allergies, infections, swelling, pain, or organ damage should be given special attention.^[51]^ With the growing popularity of electroacupuncture, the risks of electrolysis and broken needles caused by electrolysis should also be taken seriously.^[52]^ Currently, there are few reports of adverse reactions to acupuncture treatment both domestically and internationally. Additionally, the WHO has implemented the Basic Training and Safety Code for Acupuncture in 1999 to regulate the practice of acupuncture.^[53]^ This has ensured the safety and reliability of acupuncture treatment to some extent.

## 7. Conclusion

From the results of the Meta-analysis, it was seen that the effective rate and cure rate of electroacupuncture for postoperative UR in cervical cancer were better than the control group with RR values and their 95% confidence intervals of 1.32 [1.26, 1.39] and 1.92 [1.59, 2.30], respectively. The study findings indicate that indwelling catheterization, which is the most commonly used clinical intervention, did not produce satisfactory results compared to electroacupuncture. Therefore, it can be concluded that electroacupuncture is more effective in treating postoperative UR in cervical cancer. To summarize, electroacupuncture can effectively enhance the clinical efficacy of postoperative UR in cervical cancer and has advantages over conventional treatment methods, such as reducing the incidence of postoperative urinary tract infection and shortening the duration of indwelling catheterization. However, due to the limited number and quality of studies included in the Meta-analysis, further high-quality randomized controlled trials are necessary to confirm the effectiveness of electroacupuncture in promoting recovery from postoperative UR in cervical cancer.

## Acknowledgments

This research project was funded by the Natural Science Foundation of Heilongjiang Province (LH2022H080); Heilongjiang Chinese Medicine Research Project (GY202-15) No other conflicts of interest exist.

## Author contributions

**Conceptualization:** Jinlang Tan.

**Data curation:** Jinlang Tan.

**Formal analysis:** Jinlang Tan.

**Investigation:** Rui Gong.

**Methodology:** Rui Gong.

**Project administration:** Qian Zhang.

**Software:** Le Ma.

**Visualization:** Yi Zheng.

**Writing – review & editing:** Shuai Shi.
